# Effects of Neoadjuvant Chemotherapy in Ovarian Cancer Patients With Different Germline BRCA1/2 Mutational Status: A Retrospective Cohort Study

**DOI:** 10.3389/fonc.2021.810099

**Published:** 2022-01-06

**Authors:** Mengdi Fu, Chengjuan Jin, Shuai Feng, Zongyang Jia, Lekai Nie, Yang Zhang, Jin Peng, Xia Wang, Hualei Bu, Beihua Kong

**Affiliations:** ^1^ Department of Gynecology and Obstetrics, Qilu Hospital, Cheeloo College of Medicine, Shandong University, Jinan, China; ^2^ Department of Obstetrics and Gynecology, Shanghai General Hospital, School of Medicine, Shanghai Jiao Tong University, Shanghai, China; ^3^ Gynecological Oncology Department, Shandong Cancer Hospital and Institute, Shandong First Medical University and Shandong Academy of Medical Sciences, Jinan, China; ^4^ Department of Obstetrics and Gynecology, Qilu Hospital (Qingdao), Cheeloo College of Medicine, Shandong University, Qingdao, China; ^5^ Department of Radiology, Qilu Hospital, Shandong University, Jinan, China

**Keywords:** neoadjuvant chemotherapy, primary debulking surgery, BRCA, prognosis, PARPi

## Abstract

**Background:**

Whether neoadjuvant chemotherapy (NAC) followed by interval debulking surgery (IDS) against primary debulking surgery (PDS) has a differential effect on prognosis due to Breast Cancer Susceptibility Genes (BRCA)1/2 mutations has not been confirmed by current studies.

**Methods:**

All patients included in this retrospective study were admitted to Qilu Hospital of Shandong University between January 2009 and June 2020, and germline BRCA1/2 mutation were tested. Patients in stage IIIB, IIIC, and IV, re-staged by International Federation of Gynecology and Obstetrics (FIGO) 2014, were selected for analysis. All patients with NAC received 1-5 cycles of platinum-containing (carboplatin, cisplatin, or nedaplatin) chemotherapy. Patients who received maintenance therapy after chemotherapy were not eligible for this study. All relevant medical records were collected.

**Results:**

A total of 322 patients were enrolled, including 112 patients with BRCA1/2 mutations (BRCAmut), and 210 patients with BRCA1/2 wild-type (BRCAwt). In the two groups, 40 BRCAmut patients (35.7%) and 69 BRCAwt patients (32.9%) received NAC. The progression-free survival (PFS) of BRCAmut patients was significantly reduced after NAC (median: 14.9 vs. 18.5 months; p=0.023); however, there was no difference in overall survival (OS) (median: 75.1 vs. 72.8 months; p=0.798). Whether BRCAwt patients received NAC had no significant effect on PFS (median: 13.5 vs. 16.0 months; p=0.780) or OS (median: 54.0 vs. 56.4 months; p=0.323). Multivariate analyses in BRCAmut patients showed that the predictors of prolonged PFS were PDS (p=0.001), the absence of residual lesions (p=0.012), and FIGO III stage (p=0.020); Besides, PARP inhibitor was the independent predictor for prolonged OS in BRCAmut patients (p=0.000), for BRCAwt patients, the absence of residual lesions (p=0.041) and history of PARP inhibitors (p=0.000) were beneficial factors for OS prolongation.

**Conclusions:**

For ovarian cancer patients with FIGO IIIB, IIIC, and IV, NAC-IDS did not adversely affect survival outcomes due to different BRCA1/2 germline mutational status.

## Background

Ovarian cancer is the most lethal gynecological malignancy. According to the 2021 Cancer Statistics Report published by the American Cancer Society, there will be an estimated 21,410 new ovarian cancer cases and 13,770 deaths in the United States in 2021 (1). Ovarian cancer has no typical clinical symptoms in its early stage, and there is no effective screening method; therefore, the vast majority of patients have reached the advanced stage at diagnosis, leading to poor prognoses (2). For patients with International Federation of Gynecology and Obstetrics (FIGO) stage III/IV, the maximum cytoreductive surgery (R1, residual lesions less than 1 cm; R0, no residual lesions) is the most critical factor affecting the prognosis (3–5); however, if it is difficult to remove metastatic lesions in the intestine, spleen, liver or abdominal para-aortic lymph nodes due to extensive tumor metastasis, not all patients can achieve satisfactory resection from primary debulking surgery (PDS). Therefore, neoadjuvant chemotherapy (NAC) followed by interval debulking surgery (IDS) can be used as an alternative treatment to achieve maximum resection of lesions. To date, several clinical trials have confirmed that there was no significant difference between NAC-IDS and PDS in the prognoses of ovarian cancer patients (6–8).

The discovery of BRCA1/2 mutations is one of the milestones in the treatment of ovarian cancer. Ovarian cancer patients with BRCA1/2 mutations (BRCAmut), compared with BRCA1/2 wild-type (BRCAwt) patients, have a higher efficacy of platinum-based chemotherapy, a longer recurrence interval due to the presence of homologous recombination defects, and maintain a higher response rate to platinum-based chemotherapy after recurrence (9, 10). In addition, based on the synthetic lethal theory of poly (ADP‐ribose) polymerase inhibitor (PARPi), BRCAmut patients have significantly prolonged survival in salvage therapy and maintenance therapy with PARPi (11, 12). Thus, the treatment of ovarian cancer patients can be divided into two groups based on the mutational status of BRCA1/2. Whether NAC followed by IDS has a differential effect on prognosis due to BRCA1/2 mutations has not been confirmed by current studies; therefore, we conducted this retrospective study to explore the effect of BRCA1/2 mutations on neoadjuvant chemotherapy.

## Methods

### Patients and Clinical Data

**Figure 1:**
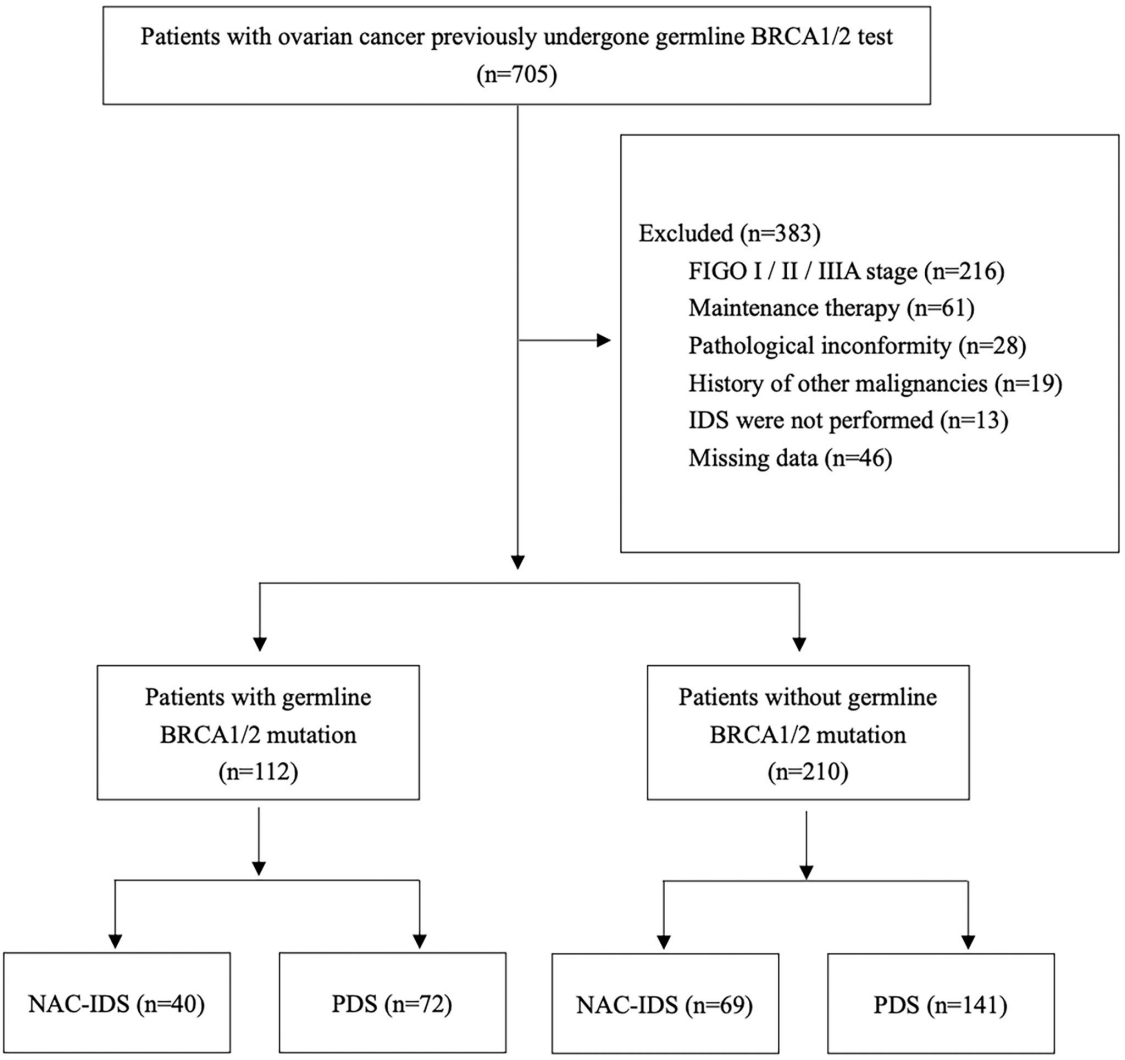
The flowchart of this study.

All patients included in this retrospective study were admitted to Qilu Hospital of Shandong University between January 2009 and June 2020. A flowchart of this study is presented in **Figure 1**. Patients were re-staged according to FIGO2014, and patients in stage IIIB, IIIC, and IV were selected for analysis. All patients with NAC received 1-5 cycles of platinum-containing (carboplatin, cisplatin, or nedaplatin) chemotherapy.

Patients who received maintenance therapy after chemotherapy, such as PARP inhibitors or bevacizumab, were not eligible for this study. The determination of response and progression-free survival (PFS) were in accordance with Response Evaluation Criteria in Solid Tumors (RECIST) 1.1 criteria (13); if the data for the RECIST criteria were not complete, CA-125 level was used as an alternative, only if the pretreatment level was at least twice the upper limit of normal (13).

Relevant medical data collected from patients included: age, the serum cancer antigen (CA) 125 level at diagnosis, maximum diameter of primary lesion, the predominant morphologic pattern of peritoneal disease of NAC patients, regimens and cycles of NAC, changes of the maximum diameter of primary lesions and sum of target lesions based on RECIST standard after NAC, hematological toxicity of NAC based on Common Terminology Criteria for Adverse Events (CTCAE) 5.0, operative duration, hemorrhage volume, residual lesions, pathological types, postoperative chemotherapy regimens, cycles and hematological toxicity, history of PARP inhibitors, FPS and OS.

For the evaluation of peritoneal disease, the main morphology were recorded as nodular or infiltrative patterns. Nodular pattern was defined as the presence of implants with predominantly well-defined borders, and infiltrative pattern was defined as the presence of implants with mostly poorly defined or infiltrative borders.

### Germline BRCA1/2 Testing

The BRCA1/2 genetic testing panels used for detection covered the entire coding sequences of the BRCA1 and BRCA2 gene, including 10–50 bases of adjacent intronic sequences of each exon. Sequencing was performed on next generation sequence (NGS) platform according to Illumina’s protocol. Sanger DNA sequencing using specific gene primers was performed to confirm each reported variant. Multiplex ligation-dependent probe amplification was used to detect BRCA 1/2 large fragment rearrangements. The variants of the mutations were classified according to the 5-class classification standard (14).

### Statistical Methods

Student’s t-test was used to compare the differences in continuous variables. The chi-square test was performed to analyze differences in clinical characteristics. PFS and OS analyses were performed by Kaplan-Meier method. Multivariate proportional odds models were used to identify variables associated with PFS outcome of BRCAmut group, and hazard ratios (HR) with 95% confidence intervals (CI) were calculated. All statistical analyses were performed by Prism 8 version 8.4.0. Significance levels were *p < 0.05, **p < 0.01.

## Results

### Characteristics of the Patients and Treatment Received

Between January 2009 and June 2020, 705 ovarian cancer patients underwent germinal BRCA1/2 gene test. A flow chart of the study was provided in **Figure 1**. There were 322 patients enrolled in the study, including 112 BRCAmut patients and 210 BRCAwt patients. All patients received carboplatin, cisplatin, or nedaplatin based chemotherapy. The chemotherapy regimens and NAC cycles were shown in **Table 1**.

**Table 1:** Chemotherapy regimens of patients.

Characteristic	BRCAmut	BRCAwt
Chemotherapy regimens of NAC		
Carboplatin based	24 (60.0%)	32 (46.4%)
Cisplatin based	11 (27.5%)	25 (36.2%)
Nedaplatin based	1 (2.5%)	2 (2.9%)
Multiple platinum	4 (10.0%)	10 (14.5%)
Cycle of NAC		
1	6 (15.0%)	10 (14.5%)
2	18 (45.0%)	28 (40.6%)
3	12 (30.0%)	21 (30.4%)
4	3 (7.5%)	7 (10.1%)
5	1 (2.5%)	3 (4.3%)
Chemotherapy regimens after surgery of NAC-IDS		
Carboplatin based	24 (60.0%)	32 (46.3%)
Cisplatin based	7 (17.5%)	15 (21.7%)
Nedaplatin based	2 (5.0%)	8 (11.6%)
Multiple platinum	7 (17.5%)	14 (20.3%)
Chemotherapy regimens after surgery of PDS		
Carboplatin based	39 (54.2%)	80 (56.7%)
Cisplatin based	15 (20.8%)	25 (17.7%)
Nedaplatin based	3 (4.2%)	4 (2.8%)
Multiple platinum	15 (20.8%)	32 (22.7%)

The characteristics of the patients were shown in **Table 2**. The median age of BRCAmut patients and BRCAwt patients were 52 years (range: 34-79) and 54 years (range: 23-75), respectively, and there was no statistical difference (p=0.439). There were also no statistical differences between the two groups in the proportion of patients receiving NAC (35.7% vs. 32.9%, p=0.606), FIGO stage (p=0.408), largest primary tumor at diagnosed (p=0.753), or serum CA-25 level (p=0.430). However, the main morphological patterns of peritoneal disease were different in patients with NAC; BRCAmut patients mainly showed nodular pattern, while BRCAwt patients were predominantly infiltrative pattern (p=0.012).

**Table 2:** Baseline characteristics of all patients.

Characteristic	BRCAmut (N =112)	BRCAwt (N = 210)	P value
Age (years)			
Median	52	54	0.439
Range	34-79	23-75	
Histologic type — no. (%)			
High-grade serous	104 (92.9%)	181 (86.2%)	NA^a^
Low-grade serous	1 (0.9%)	7 (3.3%)	
Serous not specified	5 (4.5%)	7 (3.3%)	
Mucinous	0 (0%)	4 (1.9%)	
Clear-cell	2 (1.8%)	5 (2.4%)	
Endometrioid	0 (0%)	5 (2.4%)	
Mixed	0 (0%)	1 (0.5%)	
NAC-IDS			
Yes	40 (35.7%)	69 (32.9%)	0.606
No	72 (64.3%)	141 (67.1%)	
Stage — no. (%)			
IIIB/IIIC	98 (87.5%)	190 (90.5%)	0.408
IVa/IVb	14 (12.5%)	20 (9.5%)	
Largest primary tumor at diagnosed (cm)— no. (%)			
≤5	16 (14.3%)	32 (15.2%)	0.753
>5, ≤10	57 (50.9%)	100 (47.6%)	
>10, ≤15	19 (17.0%)	46 (21.9%)	
>15	5 (4.5%)	12 (5.7%)	
Unknown^b^	15 (13.4%)	20 (9.5%)	
Serum CA-125(U/ml)			
≤1000	45 (40.2%)	98 (46.7%)	0.430
>1000	44 (39.3%)	78 (37.1%)	
Unknown^b^	23 (20.5%)	34 (16.2%)	
Peritoneal disease pattern of NAC patients			
Nodular	18 (45.0%)	21 (30.4%)	0.012^*^
Infiltrative	10 (25.0%)	38 (55.1%)	
Unknown^b^	12 (30.0%)	10 (14.5%)	

a Statistical test was not performed because of limited samples.

b These data were not included in the chi-square test.

*p < 0.05.

The baseline characteristics of the BRCAmut patients were shown in **Table 3**. Among BRCAmut patients receiving NAC-IDS and PDS, the median ages at diagnosis were 53 years (range: 34-71) and 52 years (range: 34-79), respectively, with no significant difference (p=0.988). The pathological types of patients in both groups were mainly high-grade serous carcinoma (90.0% and 94.4%), and the proportion of the maximum volume of primary lesions at diagnosis was similar. The proportion of CA125 above 1000 U/ml in patients with NAC was higher than patients with PDS (55.0% vs. 30.6%, p=0.003). The operative time of patients in the two groups were similar, with a median of 155 min (range: 80-370 min) and 155 min (range: 75-600 min), respectively (p=0.252). However, the amount of blood loss in patients receiving NAC was significantly reduced than patients with PDS (200, range 100-1000 ml vs. 400, range, 100-2000 ml; p=0.021). Moreover, NAC significantly increased the proportion of R0 excisions (55.0% vs. 27.8%; p =0.006). For adverse reactions, the most common hematologic toxicities (CTCAE ≥3) were neutrophil count decreased and white blood cell count decreased, which were 22.5% and 17.5% in the neoadjuvant patients. The proportion of postoperative hematologic toxicities in the two groups was similar (p=0.726). In addition, it is worth noting that 54.5% of BRCAmut patients were treated with PARP inhibitors in the posterior lines of treatment, with no difference between the two groups (p=0.532).

**Table 3:** Baseline characteristics of the BRCAmut patients.

Characteristic	Neoadjuvant Chemotherapy (N = 40)	Primary Debulking Surgery (N = 72)	P value
Age (years)			
Median	53	52	0.988
Range	34-71	34-79	
Histologic type — no. (%)			
High-grade serous	36 (90.0%)	68 (94.4%)	NA^a^
Low-grade serous	0 (0%)	1 (1.4%)	
Serous not specified	2 (5.0%)	3 (4.2%)	
Mucinous	0 (0%)	0 (0%)	
Clear-cell	2 (5.0%)	0 (0%)	
Endometrioid	0 (0%)	0 (0%)	
Mixed	0 (0%)	0 (0%)	
Stage — no. (%)			
IIIB/IIIC	36 (90.0%)	62 (86.1%)	0.551
IVa/IVb	4 (10.0%)	10 (13.9%)	
Largest primary tumor at diagnosed (cm)— no. (%)			
≤5	5 (12.5%)	11 (15.3%)	0.891
>5, ≤10	19 (47.5%)	38 (52.8%)	
>10, ≤15	5 (12.5%)	14 (19.4%)	
>15	1 (2.5%)	4 (5.6%)	
Unknown^b^	10 (25.0%)	5 (6.9%)	
Largest primary tumor before surgery (cm)— no. (%)			
≤5	30 (75.0%)	NA	NA
>5, ≤10	9 (22.5%)	NA	
>10, ≤15	0 (0%)	NA	
>15	0 (0%)	NA	
Unknown^b^	1 (2.5%)	NA	
Serum CA-125 (U/ml)			
≤1000	9 (22.5%)	36 (50.0%)	0.003^**^
>1000	22 (55.0%)	22 (30.6%)	
Unknown^b^	9 (22.5%)	14 (19.4%)	
Residual lesions (cm)			
0	22 (55.0%)	20 (27.8%)	0.006^**^
<1	8 (20.0%)	33 (45.8%)	
≥1	7 (17.5%)	17 (23.6%)	
Unknown^b^	3 (7.5%)	2 (2.8%)	
Duration of operation (min)			
Median	155	155	0.252
Range	80-370	75-600	
Hemorrhage of operation (ml)			
Media	200	400	0.021^*^
Range	100-1000	100-2000	
Response of NAC			
PR	23 (57.5%)	NA	NA
SD/PD	7 (17.5%)	NA	
Unknown	10 (25.0%)	NA	
History of PARPi			
Yes	21 (52.5%)	40 (55.6%)	0.532
No	17 (42.5%)	25 (34.7%)	
Unknown^b^	2 (5.0%)	7 (9.7%)	
Hematologic toxicity of NAC (≥3 CTCAE)			
White blood cell decreased	7 (17.5%)	NA	NA
Neutrophil decreased	9 (22.5%)	NA	
Anemia	1 (2.5%)	NA	
Platelet decreased	1 (2.5%)	NA	
Hematologic toxicity after surgery (≥3 CTCAE)			
White blood cell decreased	7 (17.5%)	11 (15.3%)	0.726
Neutrophil decreased	11 (27.5%)	27 (37.5%)	
Anemia	3 (7.5%)	3 (4.2%)	
Platelet decreased	1 (2.5%)	2 (2.8%)	

a Statistical test was not performed because of limited samples.

b These data were not included in the chi-square test.

*p < 0.05; **p < 0.01; NA, not available.

The baseline characteristics of the BRCAwt patients were shown in **Table 4**. The median age at diagnosis was slightly higher in BRCAwt patients in both NAC-IDS and PDS group (56 and 53 years, respectively) compared to BRCAmut patients. The proportion of FIGO stage III patients (85.5% vs. 92.9%, p=0.086) and surgical duration (median 140 vs. 155 min, p=0.484) were similar between the two groups. There was also no statistical difference in the proportion of the largest primary lesion (p=0.970). As in BRCAmut patients, the proportion of BRCAwt patients receiving NAC with ca125 level above 1000 u/ml (43.5% vs. 34.0%; p=0.021) and the R0 resection rate (43.5% vs. 28.4%; p=0.016) were higher than those in PDS patients; however, the blood loss was significantly reduced (median 300 ml, range 60-1500 ml vs. median 400 ml, range 100-6000 ml; p=0.009). The hematologic toxicity of NAC in BRCAwt patients was similar to that of BRCAmut, and there was no statistical difference in postoperative hematologic toxicity between NAC-IDS and PDS group (p=0.929). Finally, BRCAwt patients did not respond as significantly to NAC as BRCAmut; among the evaluable patients, the ratios of partial response (PR) were 46.4% (29/56) and 76.7% (23/30), respectively (p=0.025). In addition, 26.2% of BRCAwt patients were treated with PARP inhibitors in the posterior lines of treatment.

**Table 4:** Baseline characteristics of the BRCAwt patients.

Characteristic	Neoadjuvant Chemotherapy (N = 69)	Primary Debulking Surgery (N = 141)	P value
Age (years)			
Median	56	53	0.094
Range	23-75	26-73	
Histologic type — no. (%)			
High-grade serous	61 (88.4%)	120 (85.1%)	NA^a^
Low-grade serous	2 (2.9%)	5 (3.5%)	
Serous not specified	4 (5.8%)	3 (2.1%)	
Mucinous	0 (0%)	4 (2.8%)	
Clear-cell	2 (2.9%)	3 (2.1%)	
Endometrioid	0 (0%)	5 (3.5%)	
Mixed	0 (0%)	1 (0.7%)	
Stage — no. (%)			
IIIB/IIIC	59 (85.5%)	131 (92.9%)	0.086
IVa/IVb	10 (14.5%)	10 (7.1%)	
Largest tumor at diagnosed (cm)— no. (%)			
≤5	9 (13.0%)	23 (16.3%)	0.970
>5, ≤10	31 (44.9%)	69 (48.9%)	
>10, ≤15	14 (20.3%)	32 (22.7%)	
>15	3 (4.3%)	9 (6.4%)	
Unknown^b^	12 (17.4%)	8 (5.7%)	
Largest tumor before surgery (cm)— no. (%)			
≤5	42 (60.9%)	NA	NA
>5, ≤10	17 (24.6%)	NA	
>10, ≤15	4 (5.8%)	NA	
>15	2 (2.9%)	NA	
Unknown^b^	4 (5.8%)	NA	
Serum CA-125 (U/ml)			
≤1000	22 (31.9%)	76 (53.9%)	0.021^*^
>1000	30 (43.5%)	48 (34.0%)	
Unknown^b^	17 (24.6%)	17 (12.1%)	
Residual lesions (cm)			
0	30 (43.5%)	40 (28.4%)	0.016^*^
≤1	26 (37.7%)	56 (39.7%)	
>1	8 (11.6%)	38 (27.0%)	
Unknown^b^	5 (7.2%)	7 (5.0%)	
Duration of Operation (min)			
Median	140	155	0.484
Range	70-600	75-550	
Hemorrhage of Operation (ml)			
Media	300	400	0.009^**^
Range	60-1500	100-6000	
Response of NAC			
PR/CR	29 (42.0%)	NA	
SD/PD	27 (39.1%)	NA	
Unknown	13 (18.8%)	NA	
History of PARPi			
Yes	23 (33.3%)	32 (22.7%)	0.069
No	39 (56.5%)	99 (70.2%)	
Unknown^b^	7 (10.1%)	10 (7.1%)	
Hematologic toxicity of NAC (≥3 CTCAE)			
White blood cell decreased	6 (8.7%)	NA	NA
Neutrophil decreased	10 (14.5%)	NA	
Anemia	4 (5.8%)	NA	
Platelet decreased	1 (1.4%)	NA	
Hematologic toxicity after surgery (≥3 CTCAE)			
White blood cell decreased	8 (11.6%)	27 (19.1%)	0.929
Neutrophil decreased	13 (18.8%)	42 (29.8%)	
Anemia	2 (2.9%)	4 (2.8%)	
Platelet decreased	1 (1.4%)	2 (1.4%)	

a Statistical test was not performed because of limited samples.

b These data were not included in the chi-square test.

*p < 0.05; **p < 0.01; NA, not available.

### Effect of Neoadjuvant Chemotherapy on PFS and OS

**Figure 2:**
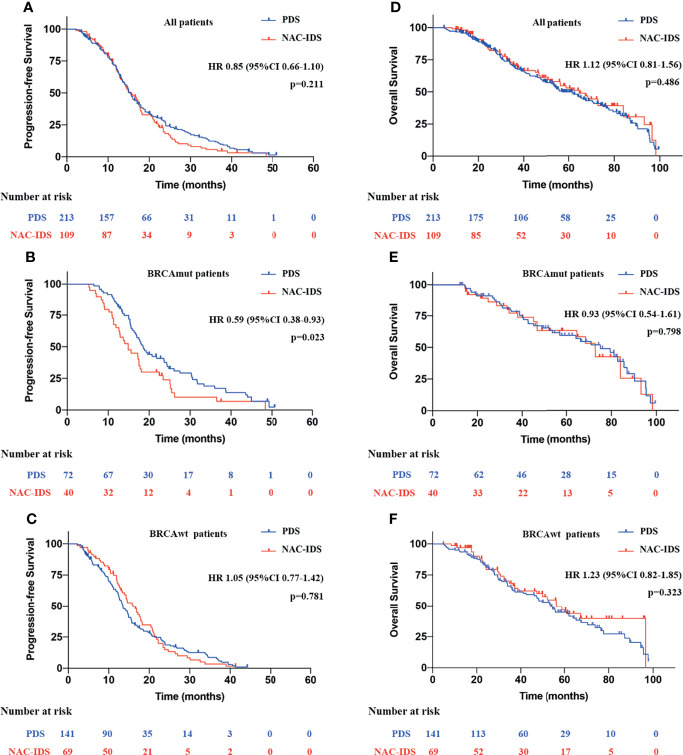
Survival analysis of patients with NAC-IDS and PDS. **(A, D)** Survival analysis of all patients included in the study. **(B, E)** Survival analysis of BRCAmut patients. **(C, F)** Survival analysis of BRCAwt patients.

The median follow-up period was 48.9 months [interquartile range (IQR), 22.8–64.2]. Regardless of the BRCA1/2 mutational status, there were no statistical differences in prognoses between patients in the NAC-IDS and PDS groups for PFS, (median: 15.4 vs. 15.6 months, HR=0.85; p=0.211) (**Figure 2A**) or OS (median: 57.1 vs. 64.7 months, HR=1.12; p=0.486) (**Figure 2D**). Further analysis found that the PFS of BRCAmut patients was significantly reduced after NAC (median, 14.9 vs. 18.5 months, HR=0.59; p=0.023) (**Figure 2B**); however, there was no statistical difference in OS (median, 75.1 vs. 72.8 months, HR=0.93; p=0.798) (**Figure 2E**). Whether BRCAwt patients received NAC had no significant effect on PFS (median, 13.5 vs. 16.0 months, HR=1.05; p=0.781) (**Figure 2C**) and OS (median, 54.0 vs. 56.4 months, HR=1.23; p=0.323) (**Figure 2F**).

Cox regression multivariate analyses were performed, with PFS and OS as the endpoint, and included the following variables: age at diagnosis, residual lesions, largest primary tumor size, FIGO stage, NAC-IDS or not, BRCA mutation, PARPi history, and CA-125 level. The strongest predictors of prolonged PFS were firstly analyzed. For all patients included in the study, the predictors were BRCA mutation (p=0.007), absence of residual lesions (p=0.003) and low CA-125 level (p=0.004); for BRCAmut patients, the predictors were FIGO III stage (p=0.020), PDS (p=0.001), and absence of residual lesions (p=0.012); for BRCAwt patients, low CA-125 level (p=0.011), absence of residual lesions (p=0.028) were predictors of prolonged PFS (**Table 5**). Further analysis of OS showed that, regardless of the patient’s other clinical characteristics, PARP inhibitor was the independent predictor in BRCAmut patients (p=0.000); For all patients in this study, PARP inhibitor and absence of residual lesions were strongest predictors of prolonged OS (PARP inhibitor, p=0.000; R0, p=0.036), and the results were consistent in BRCAwt patients (PARP inhibitor, p=0.000; R0, p=0.041) (**Table 5**).

**Table 5:** Multivariate analysis of prognostic markers related to prognoses.

Item	PFS (all patients)		PFS (BRCAmut)		PFS (BRCAwt)	
	HR (95% Cl)	*p*-value	HR (95%Cl)	*p*-value	HR (95%Cl)	*p*-value
BRCA mutation (Yes/No)	0.665 (0.493-0.859)	0.007^**^	–	–	–	–
Residual Lesions(R0/R1+R2)	0.637 (0.472-0.861)	0.003^**^	0.513 (0.305-0.864)	0.012^*^	0.653 (0.446-0.955)	0.028^*^
CA-125 (≤1000/>1000 U/ml)	0.644 (0.477-0.870)	0.004^**^	0.810 (0.469-1.397)	0.448	0.622 (0.431-0.897)	0.011^*^
FIGO (III/IV)	0.692 (0.443-1.080)	0.105	0.436 (0.217-0.877)	0.020^*^	0.780 (0.442-1.376)	0.392
NAC-IDS (No/Yes)	0.811 (0.578-1.137)	0.224	0.344 (0.186-0.637)	0.001^**^	1.086 (0.719-1.641)	0.695
Age(≤55/>55 years)	1.291 (0.956-1.743)	0.096	1.246 (0.727-2.135)	0.425	1.290 (0.898-1.854)	0.169
Maximum of Lesions (≤10/>10 cm)	1.146 (0.827-1.589)	0.413	0.677 (0.370-1.240)	0.206	1.285 (0.853-1.936)	0.230
**Item**	**OS (all patients)**		**OS (BRCAmut)**		**OS (BRCAwt)**	
	**HR (95% Cl)**	***p*-value**	**HR (95%Cl)**	***p*-value**	**HR (95%Cl)**	***p*-value**
BRCA mutation (Yes/No)	1.171 (0.788-1.739)	0.435	–	–	–	–
PARPi history (No/Yes)	0.308 (0.204-0.464)	0.000^**^	0.185 (0.088-0.387)	0.000^**^	0.336 (0.197-0.572)	0.000^**^
FIGO (III/IV)	0.673 (0.386-1.174)	0.163	0.485 (0.191-1.233)	0.129	0.752 (0.360-1.570)	0.448
NAC-IDS (No/Yes)	0.750 (0.490-1.146)	0.183	0.603 (0.277-1.311)	0.202	0.809 (0.478-1.370)	0.430
Residual Lesions(R0/R1+R2)	0.640 (0.421-0.971)	0.036^*^	0.773 (0.385-1.552)	0.469	0.572 (0.334-0.977)	0.041^*^
CA-125 (≤1000/>1000 U/ml)	0.947 (0.649-1.380)	0.775	0.777 (0.367-1.645)	0.509	1.192 (0.761-1.868)	0.442
Age (≤55/>55 years)	1.120 (0.772-1.626)	0.550	0.834 (0.412-1.687)	0.613	1.170 (0.743-1.842)	0.497
Maximum of Lesions (≤10/>10 cm)	1.150 (0.753-1.755)	0.518	0.978 (0.428-2.233)	0.958	1.242 (0.743-2.078)	0.409

*p < 0.05; **p < 0.01.

## Discussion

In this retrospective study, we found that without considering the BRCA1/2 mutation status, both PFS and OS after neoadjuvant chemotherapy followed by interval debulking surgery were similar to survivals with primary surgery followed by chemotherapy, which was consistent with the conclusions of previous randomized controlled trials (8, 15). In BRCAmut patients, NAC-IDS significantly shortened PFS, however, it had no effect on OS. For BRCAwt patients, NAC did not significantly affect the prognosis.

The proportion of patients with BRCA1/2 mutations was 35.1%, which is much higher than that previously reported in the literature (16–18). This could be related to the fact that all patients included in the study were at an advanced stage. According to previous reports, the highest proportion of BRCA1/2 mutation in Chinese ovarian cancer patients was 28.5%. However, if stage III and IV patients were separately counted, the proportion of patients with BRCA1/2 mutation was significantly increased in these studies (16, 18). Besides, another study confirmed a BRCA1/2 mutation rate of 39.2% (107/273) in ovarian cancer patients with stage IIIC-IV, which was consistent with our findings (19).

The primary evaluation criteria for the initial treatment of advanced ovarian cancer patients is whether satisfactory cytoreductive surgery can be achieved in PDS, especially R0 resection, which could significantly prolong survival (20). At present, clinical practice guidelines and expert consensus suggest that NAC is recommended for FIGO stage III to IV patients with poor physical status and unable to tolerate surgery, and for patients in whom it is difficult to achieve satisfactory tumor cytoreductive surgery (R0 and R1) (20–22). Several studies have confirmed that the serum CA-125 level was an important predictor of surgical outcome, defined as successful cytoreductive surgery with a residual tumor ≤1 cm, and is a significant factor in decision-making regarding the proper selection for PDS or NAC (23–25). In our study, the largest primary lesion at diagnosis was similar in both the NAC and PDS groups regardless of BRCA mutation, but the CA-125 levels in patients receiving NAC were significantly higher than those in the PDS group, which is one of the important reasons for choosing NAC. In addition, NAC can significantly increase the proportion of R0 resection and reduce the amount of bleeding during surgery, consistent with the conclusions of previous reports (6, 15).

Compared with BRCAwt patients, BRCAmut patients previously showed a significantly increased peritoneal tumor load (19), were associated with nodular peritoneal disease pattern (26), and showed increased sensitivity to NAC (27). In our study, we analyzed the morphological patterns of peritoneal disease of patients with NAC, and the proportion of BRCAmut patients with nodular pattern was higher than BRCAwt patients, which was consistent with the conclusion of Nougaret’s study (26); Besides, the proportion of BRCAmut patients who achieved partial response after neoadjuvant chemotherapy was 76.7%, which was significantly higher than that of BRCAwt patients (46.4%). The above reasons might cause more peritoneal lesions of BRCAmut patients to become invisible after NAC and, thus, could not be completely resected; therefore, R0 resection might not be truly achieved; instead, the complete response (CR) status was achieved through chemotherapy, which might be the main reason for reduced PFS in BRCAmut patients.

In BRCA1/2 mutated tumor cells, DNA double-stranded repair function is lost; since PARP inhibitors can block single-stranded DNA repair, this results in a “synthetic lethal” effect that leads to the death of tumor cells (9). Therefore, PARP inhibitors have excellent treatment effects in BRCAmut patients, which can significantly prolong the PFS and OS (28, 29). Further studies have confirmed that the benefit of PARP inhibitors was not limited to BRCAmut patients, but covered all ovarian cancer patients (30). The evaluation of PFS in our study, although excluding patients with PARP inhibitors for maintenance therapy, there were a large number of patients treated with PARP inhibitors in the posterior line of treatment. Through multivariate analysis, we confirmed that PARP inhibitor was the beneficial factor in both BRCAmut and BRCAwt patients for overall survival, which was the main reason for the overall survival consistency.

In previous studies affecting OS in ovarian cancer patients, R0 resection was the most critical factor (5), which was also confirmed in our study, but only applicable to BRCAwt patients. Although neoadjuvant chemotherapy reduced PFS in BRCAmut patients, PARP inhibitors were confirmed to be the independent predictor in OS analysis, which was closely related to the superior therapeutic effect of PARP inhibitors (12, 31). PARP inhibitors could also improve the prognosis of BRCAwt patients, however, the benefit was not as significant as that of BRCAmut patients (32), thus, R0 resection was still the most critical factor in these patients.

This retrospective study had several limitations. Several studies have suggested that surgery is appropriate after three cycles of NAC (6, 8, 21), patients with 1-5 cycles were included in our study. In addition, there was no detection of somatic BRCA1/2 mutation in the study, which might lead to some deviation in the results, however, the clinical characteristics of patients in BRCAmut and BRCAwt groups were similar, which could avoid the bias of the study to some extent and make the conclusion more scientific and reliable.

## Conclusion

For advanced-stage ovarian cancer patients treated with NAC followed by IDS, PFS and OS were not significantly affected in BRCAwt patients. In BRCAmut patients, NAC-IDS resulted in a shortened PFS, but had no further effect on OS, which was associated with subsequent use of PAPR inhibitors in posterior lines. NAC-IDS did not adversely affect survival outcomes due to different BRCA1/2 germline mutational status.

## Data Availability Statement

The original contributions presented in the study are included in the article/supplementary material. Further inquiries can be directed to the corresponding authors.

## Ethics Statement

The studies involving human participants were reviewed and approved by The ethics committee of Qilu Hospital of Shandong University. The patients/participants provided their written informed consent to participate in this study.

## Author Contributions

MF: Conceptualization, investigation, methodology, project administration, and writing‐original draft. CJ: Data curation, formal analysis, and software. SF, ZJ, LN, XW, and YZ: Data curation. JP: Funding acquisition, investigation and methodology. HB and BK: Formal analysis, methodology, supervision, validation, review, and editing. All authors contributed to the article and approved the submitted version.

## Funding

This work was supported by National Natural Science Foundation of China (82102963) and Department of Science Technology of Jinan city (201705051).

## Conflict of Interest

The authors declare that the research was conducted in the absence of any commercial or financial relationships that could be construed as a potential conflict of interest.

## Publisher’s Note

All claims expressed in this article are solely those of the authors and do not necessarily represent those of their affiliated organizations, or those of the publisher, the editors and the reviewers. Any product that may be evaluated in this article, or claim that may be made by its manufacturer, is not guaranteed or endorsed by the publisher.

## References

[1] Siegel RL Miller KD Fuchs HE Jemal A. Cancer Statistics 2021. CA Cancer J Clin (2021) 71(1):7–33. doi: 10.3322/caac.21654 33433946

[2] Paik ES Lee YY Lee EJ Choi CH Kim TJ Lee JW. Survival Analysis of Revised 2013 FIGO Staging Classification of Epithelial Ovarian Cancer and Comparison With Previous FIGO Staging Classification. Obstet Gynecol Sci (2015) 58(2):124–34. doi: 10.5468/ogs.2015.58.2.124 PMC4366865 25798426

[3] Chi DS Eisenhauer EL Lang J Huh J Haddad L Abu-Rustum NR. What Is the Optimal Goal of Primary Cytoreductive Surgery for Bulky Stage IIIC Epithelial Ovarian Carcinoma (EOC)? Gynecol Oncol (2006) 103(2):559–64. doi: 10.1016/j.ygyno.2006.03.051 16714056

[4] du Bois A Reuss A Pujade-Lauraine E Harter P Ray-Coquard I Pfisterer J. Role of Surgical Outcome as Prognostic Factor in Advanced Epithelial Ovarian Cancer: A Combined Exploratory Analysis of 3 Prospectively Randomized Phase 3 Multicenter Trials: By the Arbeitsgemeinschaft Gynaekologische Onkologie Studiengruppe Ovarialkarzinom (AGO-OVAR) and the Groupe D’investigateurs Nationaux Pour Les Etudes Des Cancers De L’ovaire (GINECO). Cancer (2009) 115(6):1234–44. doi: 10.1002/cncr.24149 19189349

[5] Kotsopoulos J Rosen B Fan I Moody J McLaughlin JR Risch H. Ten-Year Survival After Epithelial Ovarian Cancer is Not Associated With BRCA Mutation Status. Gynecol Oncol (2016) 140(1):42–7. doi: 10.1016/j.ygyno.2015.11.009 26556769

[6] Vergote I Trope CG Amant F Kristensen GB Ehlen T Johnson N. Neoadjuvant Chemotherapy or Primary Surgery in Stage IIIC or IV Ovarian Cancer. N Engl J Med (2010) 363(10):943–53. doi: 10.1056/NEJMoa0908806 20818904

[7] Kehoe S Hook J Nankivell M Jayson GC Kitchener H Lopes T. Primary Chemotherapy Versus Primary Surgery for Newly Diagnosed Advanced Ovarian Cancer (CHORUS): An Open-Label, Randomised, Controlled, Non-Inferiority Trial. Lancet (2015) 386(9990):249–57. doi: 10.1016/S0140-6736(14)62223-6 26002111

[8] Onda T Satoh T Saito T Kasamatsu T Nakanishi T Nakamura K. Comparison of Treatment Invasiveness Between Upfront Debulking Surgery Versus Interval Debulking Surgery Following Neoadjuvant Chemotherapy for Stage III/IV Ovarian, Tubal, and Peritoneal Cancers in a Phase III Randomised Trial: Japan Clinical Oncology Group Study JCOG0602. Eur J Cancer (2016) 64:22–31. doi: 10.1016/j.ejca.2016.05.017 27323348

[9] Tan DS Rothermundt C Thomas K Bancroft E Eeles R Shanley S. “BRCAness” Syndrome in Ovarian Cancer: A Case-Control Study Describing the Clinical Features and Outcome of Patients With Epithelial Ovarian Cancer Associated With BRCA1 and BRCA2 Mutations. J Clin Oncol (2008) 26(34):5530–6. doi: 10.1200/JCO.2008.16.1703 18955455

[10] Safra T Lai WC Borgato L Nicoletto MO Berman T Reich E. BRCA Mutations and Outcome in Epithelial Ovarian Cancer (EOC): Experience in Ethnically Diverse Groups. Ann Oncol (2013) 24(Suppl 8):viii63–8. doi: 10.1093/annonc/mdt315 24131973

[11] Ison G Howie LJ Amiri-Kordestani L Zhang L Tang S Sridhara R. FDA Approval Summary: Niraparib for the Maintenance Treatment of Patients With Recurrent Ovarian Cancer in Response to Platinum-Based Chemotherapy. Clin Cancer Res (2018) 24(17):4066–71. doi: 10.1158/1078-0432.CCR-18-0042 29650751

[12] Li N Bu H Liu J Zhu J Zhou Q Wang L. An Open-Label, Multicenter, Single-Arm, Phase II Study of Fluzoparib in Patients With Germline BRCA1/2 Mutation and Platinum-Sensitive Recurrent Ovarian Cancer. Clin Cancer Res (2021) 27(9):2452–8. doi: 10.1158/1078-0432.CCR-20-3546 33558426

[13] Rustin GJ Vergote I Eisenhauer E Pujade-Lauraine E Quinn M Thigpen T. Definitions for Response and Progression in Ovarian Cancer Clinical Trials Incorporating RECIST 1.1 and CA 125 Agreed by the Gynecological Cancer Intergroup (GCIG). Int J Gynecol Cancer (2011) 21(2):419–23. doi: 10.1097/IGC.0b013e3182070f17 21270624

[14] Plon SE Eccles DM Easton D Foulkes WD Genuardi M Greenblatt MS. Sequence Variant Classification and Reporting: Recommendations for Improving the Interpretation of Cancer Susceptibility Genetic Test Results. Hum Mutat (2008) 29(11):1282–91. doi: 10.1002/humu.20880 PMC3075918 18951446

[15] Fagotti A Ferrandina G Vizzielli G Fanfani F Gallotta V Chiantera V. Phase III Randomised Clinical Trial Comparing Primary Surgery Versus Neoadjuvant Chemotherapy in Advanced Epithelial Ovarian Cancer With High Tumour Load (SCORPION Trial): Final Analysis of Peri-Operative Outcome. Eur J Cancer (2016) 59:22–33. doi: 10.1016/j.ejca.2016.01.017 26998845

[16] Shi T Wang P Xie C Yin S Shi D Wei C. BRCA1 and BRCA2 Mutations in Ovarian Cancer Patients From China: Ethnic-Related Mutations in BRCA1 Associated With an Increased Risk of Ovarian Cancer. Int J Cancer (2017) 140(9):2051–9. doi: 10.1002/ijc.30633 28176296

[17] Wu X Wu L Kong B Liu J Yin R Wen H. The First Nationwide Multicenter Prevalence Study of Germline BRCA1 and BRCA2 Mutations in Chinese Ovarian Cancer Patients. Int J Gynecol Cancer (2017) 27(8):1650–7. doi: 10.1097/IGC.0000000000001065 28692638

[18] Bu H Chen J Li Q Hou J Wei Y Yang X. BRCA Mutation Frequency and Clinical Features of Ovarian Cancer Patients: A Report From a Chinese Study Group. J Obstet Gynaecol Res (2019) 45(11):2267–74. doi: 10.1111/jog.14090 31411802

[19] Petrillo M Marchetti C De Leo R Musella A Capoluongo E Paris I. BRCA Mutational Status, Initial Disease Presentation, and Clinical Outcome in High-Grade Serous Advanced Ovarian Cancer: A Multicenter Study. Am J Obstet Gynecol (2017) 217(3):334.e331–334.e339. doi: 10.1016/j.ajog.2017.05.036 28549976

[20] Colombo N Sessa C du Bois A Ledermann J McCluggage WG McNeish I. ESMO-ESGO Consensus Conference Recommendations on Ovarian Cancer: Pathology and Molecular Biology, Early and Advanced Stages, Borderline Tumours and Recurrent Diseasedagger. Ann Oncol (2019) 30(5):672–705. doi: 10.1093/annonc/mdz062 31046081

[21] Wright AA Bohlke K Armstrong DK Bookman MA Cliby WA Coleman RL. Neoadjuvant Chemotherapy for Newly Diagnosed, Advanced Ovarian Cancer: Society of Gynecologic Oncology and American Society of Clinical Oncology Clinical Practice Guideline. J Clin Oncol (2016) 34(28):3460–73. doi: 10.1200/JCO.2016.68.6907 PMC5512594 27502591

[22] Suh DH Chang SJ Song T Lee S Kang WD Lee SJ. Practice Guidelines for Management of Ovarian Cancer in Korea: A Korean Society of Gynecologic Oncology Consensus Statement. J Gynecol Oncol (2018) 29(4):e56. doi: 10.3802/jgo.2018.29.e56 29770626 PMC5981107

[23] Saygili U Guclu S Uslu T Erten O Demir N Onvural A. Can Serum CA-125 Levels Predict the Optimal Primary Cytoreduction in Patients With Advanced Ovarian Carcinoma? Gynecol Oncol (2002) 86(1):57–61. doi: 10.1006/gyno.2002.6719 12079301

[24] Gemer O Lurian M Gdalevich M Kapustian V Piura E Schneider D. A Multicenter Study of CA 125 Level as a Predictor of Non-Optimal Primary Cytoreduction of Advanced Epithelial Ovarian Cancer. Eur J Surg Oncol (2005) 31(9):1006–10. doi: 10.1016/j.ejso.2005.05.009 16005601

[25] Arab M Jamdar F Sadat Hosseini M Ghodssi- Ghasemabadi R Farzaneh F Ashrafganjoei T. Model for Prediction of Optimal Debulking of Epithelial Ovarian Cancer. Asian Pac J Cancer Prev (2018) 19(5):1319–24. doi: 10.22034/APJCP.2018.19.5.1319 PMC6031811 29802693

[26] Nougaret S Lakhman Y Gonen M Goldman DA Micco M D’Anastasi M. High-Grade Serous Ovarian Cancer: Associations Between BRCA Mutation Status, CT Imaging Phenotypes, and Clinical Outcomes. Radiology (2017) 285(2):472–81. doi: 10.1148/radiol.2017161697 PMC5673044 28628421

[27] Gorodnova TV Sokolenko AP Ivantsov AO Iyevleva AG Suspitsin EN Aleksakhina SN. High Response Rates to Neoadjuvant Platinum-Based Therapy in Ovarian Cancer Patients Carrying Germ-Line BRCA Mutation. Cancer Lett (2015) 369(2):363–7. doi: 10.1016/j.canlet.2015.08.028 26342406

[28] Essel KG Moore KN. Niraparib for the Treatment of Ovarian Cancer. Expert Rev Anticancer Ther (2018) 18(8):727–33. doi: 10.1080/14737140.2018.1490180 29911447

[29] Moore K Colombo N Scambia G Kim BG Oaknin A Friedlander M. Maintenance Olaparib in Patients With Newly Diagnosed Advanced Ovarian Cancer. N Engl J Med (2018) 379(26):2495–505. doi: 10.1056/NEJMoa1810858 30345884

[30] Wu XH Zhu JQ Yin RT Yang JX Liu JH Wang J. Niraparib Maintenance Therapy in Patients With Platinum-Sensitive Recurrent Ovarian Cancer Using an Individualized Starting Dose (NORA): A Randomized, Double-Blind, Placebo-Controlled Phase III Trial(). Ann Oncol (2021) 32(4):512–21. doi: 10.1016/j.annonc.2020.12.018 33453391

[31] Pujade-Lauraine E Ledermann JA Selle F Gebski V Penson RT Oza AM. Olaparib Tablets as Maintenance Therapy in Patients With Platinum-Sensitive, Relapsed Ovarian Cancer and a BRCA1/2 Mutation (SOLO2/ENGOT-Ov21): A Double-Blind, Randomised, Placebo-Controlled, Phase 3 Trial. Lancet Oncol (2017) 18(9):1274–84. doi: 10.1016/S1470-2045(17)30469-2 28754483

[32] Poveda AM Davidson R Blakeley C Milner A. Olaparib Maintenance Monotherapy in Platinum-Sensitive, Relapsed Ovarian Cancer Without Germline BRCA Mutations: OPINION Phase IIIb Study Design. Future Oncol (2019) 15(32):3651–63. doi: 10.2217/fon-2019-0343 31553234

